# Chit-Chat

**Published:** 1873-04

**Authors:** 


### Editorial Chit Chat.

New Insurance.—Louisville has a small-pox insurance company.

After Skulls.—The Emperor Francis Joseph, of Austria, finds great pleasure in collecting skulls of remarkable criminals—Where's Ruloff's ?

A New Artist.—We have a new artist in our midst, Mr. C. O. Thompson, who has executed some very fine heads in crayon,—the best we have seen.

Discontinued.—By reason of Mr. R. A. Hall's continued illness, the firm of Hall and Pratt, at Scranton, Pa., has dissolved and the business closed. Both members of this firm have hosts of friends in this city, and it is hoped they may return to us again.

Zouave Jacob.—This wonderful "medium and magnetic healer," who created such a sensation in France, a few years ago, and who was patronized by people with more money than brains, has sunk into obscurity and poverty.—Poor fellow, he seems to be deserted by those who formerly proclaimed his wonderful cures (?).

How to Construct Siamese Twins.—Rather a startling heading for an item, but this is the way a French physiologist accomplished it:—He captured two young rats, cut some skin from their sides, then joined them together, and held them there until they healed together. Don't try this plan on your babies,—it will not work so well.

The Social Club.—This is the title of a club just organized in this city, who are fitting up magnificent quarters in Stancliff block. Their furniture is of the most elegant and costly nature, and their apartments will doubtless be as beautiful as any in the city. About this time look out for scolding wives.

Central Park Museum.—The museum of Natural History at Central Park, now contains over $250,000 worth of specimens. They have just received a valuable addition to their collection,—a skeleton of a fossil deer, which was dug out of a peat bog near Dublin. It stands nine feet high, and is the largest specimen but one, known.

New Dress.—Our readers have discovered doubtless, without our referring to the fact, that the Bistoury makes its appearance in an entire new dress. The *Advertiser* Association have kindly furnished us with new materials throughout; new type, new headings, new press, and better paper. How do you like us ?

Business.—The Elizabeth, N. J., *Journal* says, that a certain undertaker there sent to one of the prominent physicians of that place, a lot of death-certificates with his card on them, as a complimentary present to the doctor. What the doctor returned to the undertaker as an acknowledgment of the compliment is not stated—likely a cathartic.

"101."—A party of benevolently inclined gentlemen of this city have formed a club for benevolent, convivial and conversational purposes, and meet at some secret chamber once a week, where, it is reported, they vote their money to some deserving charity. The club is becoming very popular, numbering among its members many of the most prominent men of the city.

The Globe Microscope.—We call the attention of students and others interested in the use of the microscope, to the advertisement of the "Globe Microscope," in this number of the Bistoury. It is really a very good little instrument, and will accomplish all that is claimed for it.—We will send one, post paid, and securely packed, for $2.75, or for ten subscribers for the Bistoury.

Barry Gray.—This gentleman, who has a host of old time friends in this city, has commenced the publication of a new magazine, "devoted to the refinements of the table. It is named *The Table.* A series of articles contributed to it by "Hadji Nicka Bauker Khan, upon Oriental Gastronomy, bears the ear marks of that chatty writer, Frank Hall, Esq., of this city. At least we will charge him with their authorship.

The New Mayor.—Major Luther Caldwell, having been elected, by a handsome majority, to rule over our city, has promised to make many needed reforms in the city government. The Major has the time, disposition and eminent capability to make a most excellent Mayor, and we predict for him a successful and popular administration, starting as he does, with the good wishes and hearty co-operation of the best men of both parties.

Rough.—A medical exchange relates the case of a Hartford physician who was summoned to attend a young man in delirium tremens. The patient had whipped out all his attendants, and the doctor thought to intimidate him by pointing a revolver at his head as he entered the room. He wouldn't "intimidate" worth a cent, but, just "went for" that doctor, seized him by the throat, choked him half to death, then kicked him down stairs.

---

Old Times.—That clerical and lay functions were once curiously conjoined, the following old-time advertisement will sufficiently show: "Wanted, for a family who have had bad health, a sober, steady person in the capacity of doctor, surgeon, apothecary, and man-midwife. He must occasionally act as butler, and dress hair and wigs. He will be required sometimes to read prayers, and to preach a sermon every Sunday. A good salary will be given."

---

A New Cure.—Some chap has written an article to a medical journal, in which he recommends saliva as a cure for rheumatism. He claims to have cured himself and numerous others, by rubbing saliva upon the parts affected. Here is an opportunity for those tobacco-chewing-loafers, whom we see upon our streets corners, to open a first-class patent medicine manufactory, bottling their vile saliva and so reaping an unexpected fortune from a credulous public. It can be done,—only try.

---

Prudent.—The very best example of prudence and forethought we have yet encountered, occured at a recent homicide trial at Columbus Ga. The physician who made an examination of the body, gave the following evidence before the jury: "Saw deceased after he was shot; he was lying upon the floor in a pool of blood, in a dying condition; I don't know how long he lived afterward; I did not make any minute examination, for the reason that I did not want you lawyers to prove that I killed him with a probe."

---

Heating the New School.—The board of education is considering the propriety of heating our new school-buildings, in the 5th district, with steam. Great difficulty has been experienced, in all our school buildings, in rendering them comfortably warm. They cannot be comortably nor healthfully heated by *any* hot-air furnace. We hope the board will adopt one of the several excellent steam heating apparatuses that have been recommended to them. We have had our say upon this subject in an article in another column, to which we refer those interested in the subject of heating and ventilation.

---

The Centennial.—Philadelphians have commenced early their preparations for the nation's celebration of its hundredth birth-day. Large meetings have already been held and fabulous sums of money subscribed, so that we may expect a grand jollification there in 1876, provided our members of Congress do not succeed in stealing the country before that time, leaving nothing to celebrate over.

Merchants, and other business men, have an announcement on their letter-heads, cards, checks, &c., "Grand Centennial Celebration here in '76." Commencing thus early they will have ample time in which to make preparations upon the magnificent scale worthy of the occasion.

---

Social Evil Hospitals.—We would like to see an hospital of this character in every city of considerable size. They have just selected a site for one in St. Louis, Mo., where, we believe, an hospital devoted exclusively to fallen women, was first proposed. They have a law in that city, by which the registration of abandoned women, their examination by a staff of physicians, and their payment of weekly and monthly dues, is enforced. Each courtesan is required to pay one dollar and a half a week, and fourteen dollars a month is required of every keeper of a house of ill-fame. This system having been inaugurated several years ago, has resulted in an accumulation of a large sum of money which is being expended in an hospital for the treatment and care of these women.

---

Grace.—It is always a pleasure to witness the graceful movements of a beautiful woman, and for this reason we have not been surprised that people of refinement and culture should visit the ballet. To see an awkward, ugly, ill-formed, and clumsy woman, attempt to ape the movements of a sprightly ballet girl, is too provoking an imposition to endure, without censuring the manager who has the impertinence to exhibit such a creature for public inspection.—The application of all this is, that Mr. E. L. Davenport, of the Chestnut St. Theatre, Phila., has placed a woman upon his stage, as leader of a ballet, who is as homely as sin, as awkward as a hippopotamus, and lacking all the sprightliness of an elephant. He calls her "M'lle Estra," who is a perfect picture of a baked apple, with clothes-pins for limbs. This puff is entirely gratuitous,—she fully merits it, for not having more discernment than the manager, and knowing that as a danseuse, she is a very conspicuous failure.

---

Hard Up.—Some one has sent us a copy of a Cincinnati paper with this advertisement marked: "Personal. Wanted. The undersigned, a healthy young man, unable to procure other employment at which he can make an honest living, desires to inform Professors of medicine and surgery, that he will submit himself to experimental operations of almost any description, for reasonable compensation. Address *Vivisection*." Now, there are several little experiments that we would like to try upon this chap, for reasonable compensation, like decapitation, for instance, so that he might inform us how long the brain remains active, after such an operation. We would be glad to know how long he could exist after removal of one or both kidney's; would like to open his stomach and witness the process of digestion, and several other little experiments of this nature. Will our friend, who was kind enough to forward the paper, please enquire upon what terms this young man could be hired, for the experiments above named?

---

A Hairy Family.—The medical press are just now interested in the description of a hairy family at Mandalay, whom the king will not permit to leave his country, for fear, likely, that they may fall into the hands of Darwin, and perhaps never be allowed to return. We have seen the following description of them, taken from a correspondence to the *London Times:* "When I was at Mandalay in '59, I saw the same woman and three of her children. The eldest and youngest were hairy, like their mother, while the second, like his father, presented no such peculiarity. The husband was a man who, report said, had been induced to wed this woman to become possessed of the marriage-portion which the king of Burmah had promised to bestow upon her on her bridal day.—The bridegroom was a plucky fellow, at any rate, though his motives may have been somewhat mercenary. The hairy woman had a pleasant, intelligent face,—there was nothing whatever repulsive in it. The hair on the face and breast was several inches long; on the forehead it was parted in the middle and blended with that of her head. Of a light brown color on her cheeks, it paled gradually toward the bridge of her nose and the centre of her lips, chin and neck."

---

Scientific Lectures.—It is a shame that those gentlemen who are fond of being considered lovers of scientific matters, and who take pride in their membership in the Academy of Sciences, should allow a scientist, like Prof. Richards, come to our city, with a magnificent apparatus for illustrating a scientific lecture, and not one of these academicans put in an appearance at the hall. We were first surprised, then chagrined at the very slender audience which greeted Prof. Richards recently, at the Opera House, upon the occasion of his lecture upon light and the sun. It was a disgrace, that a city of twenty thousand inhabitants, that furnishes a packed hall to listen to the stale jokes of Gough, or a negro minstrel band, should allow a scientist, of world-wide reputation, and of undoubted learning and skill, appear before an audience numbering less than one hundred people. We regret exceedingly that the Board of Education, did not discriminate between Prof. Richards and the usual itinerants, who clamor for admission to the presence of our pupils, by granting an adjournment of our public schools one hour earlier than usual, in order that the children might have an opportunity of witnessing the beautiful experiments in chemistry and electricity which the Professor was anxious to afford them; an entertainment that would have given them more information in one hour, than they will *ever* learn under our present facilities for teaching these sciences, in our grammar schools.

---

Imagine His Feelings.—Our friend H. is constantly relating some comical exploit of his perpetrated during his boyhood days, the most recent of which is as follows; we have not yet entirely recovered from the laughter it occasioned, but will try to relate it, stripped of the characteristic embellishments with which it was adorned by the author: When H. was a boy, his uncle presented him with a swiss musical box,—a small one, playing two or three tunes, and which, when wound up with a key, was set in motion by touching a button on the side. H. was delighted with the present, carried it about with him wherever he went, until he finally came to grief by placing it in his pocket and taking it to church with him. The sermon proved long and tedious to H., who was extremely anxious to return home and listen to the melody of his wonderful little instrment. Finally, in the midst of the sermon, an idea occurred to him,—it was a brilliant one—he thought he would just touch that button a trifle, have the instrument make a note or two, and then he'd push it back, and stop it. He did so, but the confounded thing would not stop its playing, notwithstanding he nearly wrenched the button from the box in his frantic desire to stop it. All his efforts proved unavailing,—the machine kept on playing, to the great surprise of the congregation and the supreme disgust of the officiating clergyman. All eyes were turned upon poor H., who would have given the world, including the musical box, could the floor have opened and let him through, out of sight. All tragedies have an ending, and it came at last to H., who was collared by the sexton and marched down through the long aisle of the church, the infernal box playing Yankeedoodle, in time to every step. H. says, when he reached the door, he fainted; but the very first thing he did, when restored to conciousness, was to mash the confounded musical box.

